# Association between mortality from suicide in England and antidepressant prescribing: an ecological study

**DOI:** 10.1186/1471-2458-4-63

**Published:** 2004-12-21

**Authors:** Oliver WC Morgan, Clare Griffiths, Azeem Majeed

**Affiliations:** 1 Health and Care Division, Office for National Statistics, 1 Drummond Gate, London, United Kingdom; 2 Department of Primary Care and Social Medicine, Imperial College, Charring Cross Hospital, London, United Kingdom

## Abstract

**Background:**

Antidepressant prescribing has been increasing in England. Studies in other countries suggest that while this may be associated with reduced suicide rates, it may also be associated with increased fatal poisoning from antidepressant drugs. We therefore conducted an ecological study to assess the association between prescription rates for antidepressants and suicide or fatal antidepressant-related poisoning in England.

**Methods:**

The Office for National Statistics provided information on the number of suicides, antidepressant-related poisoning deaths and populations for England between 1993 and 2002. The Department of Health supplied data on prescriptions for all antidepressants dispensed in England. Associations between prescriptions and deaths were assessed using Spearman's rank correlation coefficient.

**Results:**

There were 46,747 suicides, 3,987 deaths involving tricyclic antidepressants and 430 involving selective serotonin re-uptake inhibitors and other antidepressants. Increased antidepressant prescribing was statistically associated with a fall in suicide rates (Spearman's r_s _= -0.73, p = 0.02) and fatal poisoning involving tricyclic antidepressants (r_s _= -0.64, p = 0.05). In contrast, increased prescribing of selective serotonin re-uptake inhibitors and other antidepressants was statistically associated with an increase in fatal poisoning involving these drugs (r_s _= 0.99, p < 0.001).

**Conclusion:**

Increased prescribing of antidepressants may indicate improved diagnosis and treatment of depression in primary care. Our analysis suggests that this was accompanied by lower suicide rates. A decrease in poisoning deaths involving tricyclic antidepressants may suggest a change in preference for using serotonin reuptake inhibitors and other antidepressant drugs for high-risk patients. This may also partially explain the increase in deaths involving these drugs. Due to the ecological nature of the design, we cannot say conclusively whether reduced suicide rates are a direct consequence of increased antidepressant prescribing rates. To confirm these associations, individual level data on prescribing and suicide is needed.

## Background

Around 5000 people take their own lives in England every year [1]. The Government's White Paper *Saving Lives: Our Healthier Nation *sets a target to reduce suicide deaths by 20% by 2010 [2]. One of the most common risk factors amongst suicide victims is a major depressive episode (present in about 60–90% of victims) [3]. Treatment of depressed patients can reduce suicide risk by half [3]. Hence, increased diagnosis and treatment of depression in primary care is a key element in reducing suicide risk.

Studies in Sweden [4], Denmark [5], Finland [5], Norway [5] and Australia [6] suggest that increased used of antidepressants drugs is related with lower suicide rates. However, other studies have also reported a positive association between increased prescribing of antidepressants and higher death rates from antidepressant overdose in Finland [7], Norway [8], Australia [9] and England [10]. In contrast, studies in Italy [11], Austria [12] and Ireland [13] have not observed increased suicide or fatal antidepressant poisoning following increases in antidepressant prescribing.

In England, prescriptions for antidepressants have been increasing [14,15]. We conducted an ecological study to assess trends in prescription rates for antidepressants in England and rates for suicide and fatal antidepressant-related poisoning.

## Methods

### Suicide

Suicide deaths were defined as deaths where the coroner has given a verdict of suicide or where an open verdict was reached in a death from injury or poisoning. This is because it is thought that most open verdicts are cases where the harm was self-inflicted but there was insufficient evidence to prove that the deceased deliberately intended to kill themselves [16]. Open verdicts account for about 30% of male and 40% of female suicide deaths [16]. Suicide deaths were identified using the International Classification of Diseases codes shown in Table 1.

**Table 1:** ICD-9 and ICD-10 classification for suicides

**Description**	**ICD-9**	**ICD-10**
All suicides	E950–E959, E980–E989 excluding E988.8 with verdict pending	X60–X84, Y10–Y34 excluding Y33.9 with verdict pending
Non-drug poisoning suicide	As above excluding E950.0–E950.5 and E980.0–E980.5	As above excluding X60–X64 and Y10–Y14

### Antidepressant-related deaths

The Office for National Statistics has stored drug-poisoning mortality data for England and Wales from 1993 onwards in a dedicated database [17]. The database contains data on cause of death, individual characteristics (age and sex) as well as textual information from the death certificate. This textual information has been examined to identify and code the substances involved in the death. All drugs mentioned are also coded to British National Formulary categories where appropriate.

Drug poisoning deaths were defined using the International Classification of Diseases codes shown in Table 2. Antidepressant-related deaths were defined as any drug poisoning death where an antidepressant drug was mentioned on the death certificate, with or without mentions of alcohol or other drugs. Antidepressant drugs were further classified according to their BNF categories (Table 3).

**Table 2:** ICD-9 and ICD-10 classification for drug poisoning

**Description**	**ICD-9**	**ICD-10**
Mental and behavioural disorders due to drug use (excluding alcohol and tobacco)	292, 304 305.2-9	F11–F16, F18–F19
Accidental poisoning by drugs, medicaments and biological substances	E850–E858	X40–X44
Intentional self-poisoning by drugs, medicaments and biological substances	E950.0–E950.5	X60–X64
Poisoning by drugs, medicaments and biological substances, undetermined intent	E980.0–E980.5	Y10–Y14
Assault by drugs, medicaments and biological substances	E962.0	X85

**Table 3:** British National Formulary Categories for Antidepressant Drugs

**BNF Category**	**Description**
4.3.1	Tricyclics and related antidepressants
4.3.2	Monoamine oxidase inhibitors
4.3.3	Selective serotonin re-uptake inhibitors
4.3.4	Other antidepressants

### Prescriptions for antidepressant drugs

The Department of Health supplied data on prescriptions for all antidepressants dispensed in England between 1993 and 2002. Prescription information is derived from the Prescription Cost Analysis (PCA) system, which collects data from all prescriptions dispensed in the community [18]. This includes community pharmacists, dispensing doctors and prescriptions submitted by prescribing doctors for items personally administered (i.e. given by the doctor during a consultation). PCA data also includes prescriptions written in Wales, Scotland, Northern Ireland and the Isle of Man but dispensed in England. Drugs dispensed in hospital or private prescriptions are not included.

### Analysis

Directly age-standardised rates for suicide and antidepressant-related deaths were calculated using the European Standard Population. Populations for England between 1993 and 2002 were interim revised estimates published by ONS in September 2003. Prescription rates were presented as number of prescriptions per 100 population. Statistical associations between prescription rates and directly age-standardised rates for antidepressant-related poisoning deaths and suicides were assessed using Spearman's rank correlation coefficient.

## Results

In England between 1993 and 2002, there were 46,747 suicides. Suicide was twice as common in men as in women. There were 3,987 deaths involving tricyclic antidepressants (TCAs, BNF 4.3.1,) and 430 involving selective serotonin re-uptake inhibitors and other antidepressants (SSRIs & others, BNF 4.3.3 & 4.3.4). The number of antidepressant-related deaths was similar for men and women. Changes in age-specific rates were similar across all age groups for suicide and antidepressant-related deaths.

Between 1993 and 2002, age-standardised mortality rates for suicide decreased from 98.2 to 84.3 per million population (Table 4). Rates for TCA poisoning also decreased from 8.6 to 5.3 per million while mortality rates for SSRIs & others increased from 0.2 to 1.8 per million. During the same period, prescriptions per 100 population for all antidepressants increased almost two and a half times from 22.4 to 53.2 per 100 population (Table 4). While there was a modest increase for TCAs from 17.5 to 19.9 per 100 population, prescriptions for SSRIs and others increased more than seven-fold from 4.6 to 33.1 per 100 population.

**Table 4:** Age-standardised mortality rates for suicide and antidepressant-related poisoning deaths and prescriptions per 100 population in England

**Year**	**Age-standardised mortality rates per million population**			**Prescriptions per 100 population**		
		
	**Suicide**	**TCAs**	**SSRIs**	**All**	**TCAs**	**SSRIs**
1993	98.2	8.6	0.2	22.4	17.5	4.6
1994	94.8	8.7	0.1	24.5	17.9	6.2
1995	95.6	8.7	0.4	27.4	18.3	8.7
1996	90.6	9.7	0.4	30.9	18.8	11.8
1997	92.8	9.4	0.6	34.7	19.3	15.1
1998	95.7	8.8	0.7	37.9	19.7	17.9
1999	95.1	8.0	1.1	41.2	19.7	21.2
2000	90.2	6.9	1.3	44.9	19.7	25.0
2001	84.9	5.8	1.8	49.3	19.8	29.3
2002	84.3	5.3	1.8	53.2	19.9	33.1

Note: Directly standardised to the European Standard Population

Spearman's rank correlation indicate that rates for all suicides were inversely related to prescribing rates for all antidepressants combined, r_s _= -0.73, p = 0.016 (Table 5). Mortality rates for non-drug poisoning suicides showed a strong statistical association with prescription rates for all antidepressants, r_s _= -0.89, p = 0.005. Antidepressant-related poisoning mortality rates were also inversely statistically associated with prescription rates for all antidepressants combined, but the association was much weaker, r_s _= -0.45, p = 0.187. As most antidepressant-related poisoning deaths were due to TCAs, the relationship between TCA deaths and prescribing were similar to all antidepressants combined. In contrast, SSRI and other-related deaths increased and prescriptions for SSRIs & others both increased during the study period r_s _= 0.99, p < 0.001.

**Table 5:** Spearman's rank correlation coefficients and p-values for directly age-standardised suicide and antidepressant poisoning rates and prescription rates, England 1993 to 2002

**Cause of death**	**Prescriptions**	**r_s_**	**p**
All suicides	All antidepressants	-0.73	0.016
Non-drug poisoning suicide	All antidepressants	-0.89	0.005
Antidepressant-related poisoning deaths	All antidepressants	-0.45	0.187
Tricyclic antidepressant -related poisoning deaths	Tricyclic antidepressants	-0.64	0.05
Selective serotonin re-uptake inhibitor and other antidepressant-related poisoning deaths	Selective serotonin re-uptake inhibitors and other antidepressants	0.99	<0.001
			
Antidepressant only poisoning deaths*	All antidepressants	-0.53	0.12
Tricylclic antidepressant only poisoning deaths*	Tricyclic antidepressants	-0.71	0.022
Selective serotonin re-uptake inhibitor and other antidepressant only poisoning deaths*	Selective serotonin re-uptake inhibitors and other antidepressants	0.94	<0.001

* Where no other drugs were mentioned on the death certificate

A previous analysis found that about a third of antidepressant-related deaths also have other drugs or substances mentioned on the death certificate (26% of deaths involving TCAs, 72% involving SSRIs and others) [19]. Hence, death certification data may over-estimate deaths attributable to antidepressant overdose. When we excluded deaths involving other substances from our analysis, antidepressant mortality rates were more closely related with antidepressant prescribing (TCAs r_s _= -0.71, p = 0.022, SSRIs & others r_s _= 0.94, p < 0.001).

## Discussion

Increased prescribing of antidepressants was statistically associated with reduced suicide mortality rates. TCA-related poisoning deaths also decreased during the study period although the evidence for a statistical association with prescribing rates was weaker. Increased prescribing of SSRIs and other antidepressants was statistically associated with increased mortality rates involving SSRI & other-related poisoning, although the rates were much smaller than for TCA-related poisoning or suicide. Because our study uses population level data, we cannot conclude that these associations are necessarily causal.

### Limitations

Although the ONS drug poisoning database is the most complete record of drug poisoning statistics available, about 10% of these deaths have no specific information about drug(s) taken [1]. Inconsistency in the investigation and recording of drug-poisoning deaths may mean that not all antidepressant-related deaths are identified [17]. Prescribing Cost Analysis data do not record why the drug was prescribed and antidepressants are increasingly used for conditions other than depression [20]. This may lead to an over-estimation of their use in the treatment of depression. Furthermore, prescribing data does not provide information on the age or sex of the patient. Both of these variables are likely to be associated with prescribing and suicide or poisoning, and may have introduced confounding into our analysis.

Further bias may have been introduced due to prescriber and patient factors. Doctors may perceive SSRIs and other antidepressants to have clinical advantages over TCAs due to their lower toxicity, possibly making them more popular for treating newly diagnosed (an uncontrolled) depression [21], treating individuals who are at a greater risk of overdose [22] or individuals who have not previously responded to treatment.

### Interpretation

Assessing the impact of increased antidepressant prescribing is difficult. This is because antidepressants are both a treatment and method for suicidal behaviour. Suicide is also closely linked to availability of methods [23], generating concern that increased prescribing of antidepressants may increase fatal poisoning rates. There has also been concern that some antidepressants, particularly the SSRIs, may actually precipitate suicide behaviour [24]. In a recent article, Jick et al suggested that both older and newer antidepressants were associated with 3–4 fold increased risk of suicidal behaviour in the first month after starting treatment [25]. Hence, determining the balance of risk and benefit of treatment with antidepressants is not straightforward. Using a theoretical model based on paediatric trails of antidepressants drugs and increased prevalence of suicidal thoughts and self-harm, Gunnell and Ashby suggest that compared to 1991, antidepressants may have contributed to an excess of 388 suicides (95% credibility interval -202 to 704) in 2002 [26].

However, our study indicates that at a population level, increased antidepressant prescribing in England was statistically associated with lower rates of suicide and antidepressant-related poisoning. One explanation for our findings is that SSRIs and other antidepressants are being used in preference to TCAs to treat depression. The perceived side effects of TCAs means they are often prescribed or taken at sub-therapeutic doses [21,27], leading to poor management of depression and increased risk of suicide [28,29]. Furthermore, the lower toxicity profiles of the newer antidepressants may also lead to preferential use of SSRIs for high-risk patients, which may in turn explain the increase in deaths associated with SSRIs and other antidepressants. Some support for this is provided by a previous analysis of antidepressant-related deaths during the same study period, which showed a decrease in antidepressant-related deaths per million prescriptions for all TCAs [19].

Nevertheless, the clinical benefits of antidepressant drugs for the management of depression and prevention of suicide are still unclear. A recent Cochrane review found little difference between antidepressants and active placebos on improvement of mood [30]. (Active placebos contain a drug, which is not thought to have an effect on the disorder being treated, but which mimics the effect of taking an active substance). The possibility that antidepressants have limited effectiveness on treatment of depression suggests that we should be cautious when drawing conclusions about the relationship between antidepressant prescribing and suicide. Increased antidepressant prescribing may be a marker of improved diagnosis and treatment of depression in primary care with greater use of non-pharmacological and psychosocial interventions [6]. Alternatively, suicide rates may reflect other trends during the study period: unemployment, which is associated with suicide [31], fell from about 10% to below 5% during the study period [32]. In contrast, consumption of alcohol, mentioned in 28% of antidepressant poisoning deaths, remained relatively stable [33]. Hence, the ecological nature of our data means that we cannot say conclusively whether reduced suicide rates is a direct consequence of increased antidepressant prescribing rates.

## Conclusions

The public health impact of increased prescribing of antidepressants remains unclear, including whether this leads to lower suicide rates, and whether this could be a marker for improved diagnosis and treatment of depression in primary care. Our analysis of data for England between 1993 and 2002 suggest that increased prescribing of antidepressants was associated with lower suicide rates and probably lower rates of poisoning involving TCAs. This may have been due to a change in preference for SSRIs and other antidepressants for high-risk patients but also to other secular trends in areas like unemployment. The increase in prescribing rates does however probably explain the increase in fatal poisonings involving SSRIs and other antidepressants, which are generally considered to be less toxic than older agents. Further analysis of individual level data, possibly from longitudinal general practice patient data as well as from primary care based clinical trials, are needed to provide better evidence of the role of antidepressants in reducing suicide.

## Competing interests

The author(s) declare that they have no competing interests.

## Authors' contributions

OM designed the study, conducted the analysis and drafted the manuscript. CG participated in conducting the analysis and drafting the manuscript. AM participated designing the study and drafting the manuscript. All authors read and approved the final manuscript.

## Pre-publication history

The pre-publication history for this paper can be accessed here:


